# Topically Applied Connective Tissue Growth Factor/CCN2 Improves Diabetic Preclinical Cutaneous Wound Healing: Potential Role for CTGF in Human Diabetic Foot Ulcer Healing

**DOI:** 10.1155/2015/236238

**Published:** 2015-02-18

**Authors:** F. R. Henshaw, P. Boughton, L. Lo, S. V. McLennan, S. M. Twigg

**Affiliations:** ^1^Sydney Medical School and Charles Perkins Centre, University of Sydney, Sydney, NSW 2006, Australia; ^2^Department of Biomedical Engineering, School of Aerospace, Mechanical and Mechatronic Engineering, University of Sydney, Sydney, NSW 2006, Australia; ^3^Department of Endocrinology, Royal Prince Alfred Hospital, Camperdown, NSW 2050, Australia

## Abstract

*Aims/Hypothesis*. Topical application of CTGF/CCN2 to rodent diabetic and control wounds was examined. In parallel research, correlation of CTGF wound fluid levels with healing rate in human diabetic foot ulcers was undertaken. *Methods*. Full thickness cutaneous wounds in diabetic and nondiabetic control rats were treated topically with 1 *μ*g rhCTGF or vehicle alone, on 2 consecutive days. Wound healing rate was observed on day 14 and wound sites were examined for breaking strength and granulation tissue. In the human study across 32 subjects, serial CTGF regulation was analyzed longitudinally in postdebridement diabetic wound fluid. *Results*. CTGF treated diabetic wounds had an accelerated closure rate compared with vehicle treated diabetic wounds. Healed skin withstood more strain before breaking in CTGF treated rat wounds. Granulation tissue from CTGF treatment in diabetic wounds showed collagen IV accumulation compared with nondiabetic animals. Wound *α*-smooth muscle actin was increased in CTGF treated diabetic wounds compared with untreated diabetic wounds, as was macrophage infiltration. Endogenous wound fluid CTGF protein rate of increase in human diabetic foot ulcers correlated positively with foot ulcer healing rate (*r* = 0.406; *P* < 0.001).* Conclusions/Interpretation*. These data collectively increasingly substantiate a functional role for CTGF in human diabetic foot ulcers.

## 1. Introduction

Foot ulceration secondary to diabetes occurs in up to one-quarter of people with diabetes [1] and is the commonest cause of lower limb amputation, accounting for 50–70% of nontraumatic cases [2]. Diabetes increases the risk of lower extremity amputation by 10 to 20 times [3, 4]. The estimated cost to the US healthcare system of diabetic foot ulceration and related amputations is more than $10·9 billion [5]. Thus diabetic foot ulceration is a cause of significant morbidity and financial burden.

Wounds in diabetic patients typically show abnormal healing, characterized by chronicity, persistent inflammation, copious exudate, hypergranulation, increased bacterial load, and reduced ability to heal [6]. This delayed healing is thought to be due to a combination of factors including macro- and microvascular disease, neuropathy, bacterial infection, local pressure due to foot deformity, and the adverse local metabolic environment caused by diabetes. Furthermore, pathogenic factors lead to suboptimal extracellular matrix (ECM) composition: it has been hypothesized that a cytokine/chemokine mediated imbalance between synthetic and degradative matrix pathways is responsible for the reduced amount and quality of ECM [7]. Indeed, human diabetic wounds exhibit an excess of proinflammatory cytokines such as TNF-*α*, which contribute to an environment of increased protease activity in diabetic wounds [7].

CTGF is a 32–38 kDa member of the CCN family, a group of proteins which share a common modular structure [8]. Also known as CCN2, CTGF is able to stimulate fibroblast proliferation and differentiation in sites including in skin, thus enhancing ECM production [9]. CTGF is overexpressed in many profibrotic conditions such as scleroderma [10]. CTGF is able to promote cell adhesion and is chemotactic for inflammatory cells especially macrophages; it is mitogenic and also assists in cell differentiation [11, 12].* In vivo*, CTGF application accelerated wound healing in a monkey burns model [13].

Changes in CTGF gene expression and protein levels have been reported in some tissues and biological fluids of diabetic subjects, especially where fibrosis occurs to relative excess [14]. This includes diabetic nephropathy [15], diabetic cardiomyopathy [16], and retinopathy [17] where CTGF is elevated. In contrast, in skin, in nonhuman primate (Baboon) studies we have recently shown that intact CTGF protein is deficient in diabetic wound tissue compared with wound tissue in nondiabetic animals [18]. In that study the wound inflammatory and protease environment was also increased in diabetes and CTGF protein accumulation in wounds was delayed. To date however, CTGF regulation in human diabetic wounds has not yet been reported and the effect of topical application of CTGF to diabetic wounds has not been described in any animal model. The aims of this study were to (i) examine if topical rhCTGF improves wound healing in a well defined model of diabetic rodent cutaneous wounding and (ii) determine whether CTGF increase in wound fluid from human diabetic foot ulcers demonstrates a relationship with wound healing rate.

## 2. Methods and Materials

### 2.1. Connective Tissue Growth Factor (CTGF) and Its Application

Recombinant human CTGF (rhCTGF) was expressed utilizing adenovirus in 911 cells and purified and quantitated in house exactly as previously described, with confirmation of its ECM inducing bioactivity [19].

### 2.2. Induction of Diabetes and Creation of Skin Wounds

Male Sprague-Dawley rats (*n* = 52) purchased from Australian Laboratory Supply (Perth, Australia), aged between 6 and 7 weeks, were used in these studies. Ethics approval for induction of diabetes in animals and creation of wounds was obtained from the Animal Research Ethics Committee, Sydney South Western Area Health Service (SSWAHS). Type 1 diabetes was induced in 23 animals using streptozotocin (STZ: 65 mg/kg, Calbiochem, Sydney, Australia) and animals were maintained on 2–4 IU of insulin (Mixtard, Novo-Nordisk) every second day to prevent weight loss and ketoacidosis. After 7 weeks, diabetic (*n* = 23) and control (*n* = 29) animals were anesthetized using Ketamine (85 mg/kg Pfizer, Sydney, Australia) and Xylazine (5 mg/kg, Bayer, Leverkusen, Germany). The dorsal skin was prepared for wounding by shaving with clippers and depilation (Nads, Baulkham Hills, Australia) and swabbing with antiseptic (Betadine, Symbion, Melbourne, Australia). As we previously described [20], 4 full thickness dorsally placed excisional wounds per rat were then created using an 8 mm punch biopsy (Stieffel Laboratories, NSW, Australia). The wounds included the panniculus carnosus and exposed the underlying dorsolateral skeletal muscle fascia. At the time of wounding all animals were treated with a single dose of parenteral antibiotic (Ampicillin: 50 mg/kg).

### 2.3. Topical RhCTGF Treatment of Skin Wounds

RhCTGF as 1 *μ*g in 20 *μ*L of sterile phosphate buffered saline (PBS) was applied topically to two of the ulcers in each animal and 20 *μ*L PBS was applied topically to the remaining two wounds. Ulcers were each occluded using a transparent dressing (Tegaderm, 3M, NSW, Australia) which was secured peripherally using Hypafix tape (Smith and Nephew, Victoria, Australia). At an interval of 24 h animals were anaesthetized in the manner already described and treated with a second dose of rhCTGF, or PBS to the same wounds, as before. Wounds were again occluded using Tegaderm and Hypafix.

In one series of experiments, the effect of rhCTGF on wound closure was determined by tracing the circumference of the wounds onto transparencies on the day of wounding and then at regular intervals thereafter (Tegaderm dressing packaging). In a parallel series, rats from each group were terminated at days 7 and 14 and the tissue containing the wound was excised and either (i) fixed in paraformaldehyde (4% in PBS) for later immunohistochemical analysis or (ii) snap-frozen in liquid nitrogen for determination of wound breaking strength.

### 2.4. Analysis of Wound Closure

Wound circumference tracings were translated into computer images using pen-tablet software (Bamboo, Wacom, USA) for quantification of wound area using Image J (Research Services Branch, NIMH, USA) [20]. Wound closure was then calculated and results are expressed as a percentage of original wound size.

### 2.5. Analysis of Wound Breaking Strength

Wound breaking strength was determined using our published methods [18], adapted for rodents. Wounds that had been excised on day 14 were removed from frozen storage and cut into shape using an aluminum template. Width, length, and thickness of skin pieces were measured using calipers. The tissue was allowed to thaw and tensile strength was determined at room temperature. Tissue ends were securely mounted on a 2 mm balsa wood sandwich coupling using cyanoacrylate and placed in the jaws of an Elf3400 Tensiometer (BOSE EnduraTec, Minnetonka, MN, USA). Load and displacement until the time of skin rupture at the healed wound site were obtained using a 45 N load at a cross-head speed of 10 mm/min. Cross-sectional area was determined from original skin thickness measurements and values were used to calculate stress and strain and Young's modulus (tensile strength).

### 2.6. Analysis of Wound Cellular Content and Extracellular Matrix

The excised paraformaldehyde embedded wounds were sectioned (5 *μ*m/section) perpendicular to the wound surface. Macrophages from sections obtained at days 7 and 14 were identified by immunohistochemical staining with CD68 (Abcam, Cambridge, MA) as described previously [20]. In brief, following antigen retrieval, nonspecific binding was blocked by incubation in 10% v/v goat serum and, after washing with PBS, the sections were incubated for 60 min. in CD68, (1 : 200) mouse IgG1. A further PBS wash was carried out before incubation with 1 : 400 dilution of secondary antibody, (Biotinylated anti-mouse). Cells were visualized using peroxidase-conjugated secondary antibodies and 3,3′-diaminobenzidine chromogen (Vector Laboratories, Burlingame, CA). The number of macrophages in 20 sequential fields was determined (at ×1000 magnification) by a single observer blinded to animal grouping and treatment status.

Fibroblast and endothelial cells from sections obtained at days 7 and 14 were identified by immunohistochemical staining using anti-*α*-smooth muscle actin (*α*-SMA) primary antibody 1 : 200 (*α*-SMA, Abcam, Burlingame, CA) and their characteristic morphology. Detection then involved secondary antibody and the ABC method as described for CD68 staining above and as per [20]. Cells were stained and visualized as described for macrophages. The *α*-SMA positive fibroblasts and endothelial staining intensity within each wound section was scored by two independent observers, each blinded to animal grouping and treatment status. As previously described, the scoring was based on a grade of 0–3 where zero was for no staining, up to three for intense staining, and any background staining in the isotype control section was subtracted from the overall staining score [18, 21].

Immunohistochemical staining for collagen IV was performed as previously described using the goat generated anti-collagen IV polyclonal antibody Abcam (Ab86042) at 1 : 200 titer as the primary antibody. The subsequent staining and detection method was that previously used in diabetic preclinical studies [18, 21], with the same scoring method as described for *α*-SMA quantitation above.

### 2.7. Human Diabetic Foot Ulcer Wound Fluid Analysis

In a separate series of experiments relative CTGF levels in postdebridement wound fluid in diabetic wounds were determined in patients managed at the multidisciplinary high risk foot service, where all aspects of diabetic foot care had been optimized [22, 23] (Table 3). There were 32 serial subjects with type 2 diabetes and each had dense peripheral neuropathy, and some were also ischemic ulcers (Table 1). Ethics approval for this study was obtained from the Human Research Ethics Committee, Sydney South Western Area Health Service.

Wound fluid was obtained following debridement using a sterile 1 cm^2^ Whatman 4 mm filter paper as previously described [22, 23]. The protein concentration of the wound fluid was determined using the BioRad protein assay (BioRad, Sydney Australia) and a sample containing a standard amount of total protein (30 *μ*g) was applied to a 12.5% SDS-PAGE under reducing conditions, as previously described [18, 24]. Western immunoblot analysis using an in-house generated anti-CTGF polyclonal primary antibody (196) at 1 : 1,000 titer and subsequent goat anti-rabbit secondary antibody was undertaken using standard methods [18]. Data determined by densitometry was then analyzed and presented as % change in immunoreactive CTGF in the wound fluid compared with % ulcer healing rate, across the same time interval. In most cases, each wound had 3 CTGF measures, spread in total across 45 or more days.

### 2.8. Statistical Analysis

Wound data is expressed as mean ± SD or mean ± SEM, each as indicated. Analysis was performed by one way ANOVA, with post hoc correction by Bonferoni's multiple comparison test, or by unpaired *t*-test, each as indicated. A *P* value < 0.05 was considered statistically significant. Simple linear correlation analysis was performed for the human CTGF % change in postdebridement wound fluid data in relation to time.

## 3. Results

### 3.1. Effect of CTGF on Wound Closure

Group data for macroscopic wound closure measurement, expressed as a percentage change from the initial wound area at respective time points, is shown in Table 1. Diabetic animals (D + PBS) were found to have wound closure rates that were slower than those in control (nondiabetic) animals (C + PBS), with this being significant on day 7. In addition, macroscopic wound closure was significantly enhanced on days 7, 10, and 14 in the CTGF treated diabetic group compared with the nonactively treated diabetic group with vehicle alone (D + PBS), Table 1. In contrast to CTGF effects in diabetic wounds, nondiabetic wounds treated with CTGF (C + CTGF) did not show improved closure rates relative to controls alone (C + PBS).

### 3.2. CTGF Therapy and Wound Breaking Strength

The effect of CTGF on the wound breaking strength at the wound site was also measured. Analysis by ANOVA showed that untreated diabetic wounds elongate less well before breaking than either treated or untreated control wounds (*P* < 0.05) (average 2.20 MPa compared with 3.08 and 3.45 MPa, resp.), indicating that diabetic wounds are functionally less flexible (Table 2). CTGF treated diabetic wounds had a slightly higher mean final strain (2.32 MPa) than the untreated diabetic wounds (2.20 MPa); however this did not reach statistical significance. Young's modulus which measures stress in relation to strain and thus wound elasticity was not significantly different between groups in this model.

### 3.3. Effect of CTGF Treatment on Wound Cellular Content and Matrix

**Figure 1:**
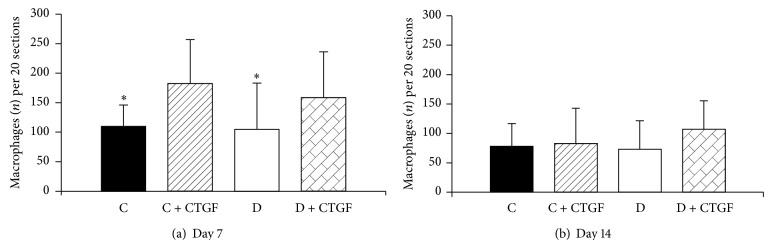
Wound macrophage counts. (a) CTGF treated (control and diabetic groups combined) is different to CTGF untreated (control and diabetic groups combined); ^*^ *P* < 0.05 by unpaired *t*-test at day 7. No statistical difference was observed between groups at day 14 (b). Results are expressed as mean ± SEM.

Macrophage cell counts in CD68 stained sections (Figure 1) were lower in PBS alone treated animals compared with CTGF treated animals at day 7. Macrophage cell count declined in both treated and untreated groups by day 14, compared with day 7. Interestingly the macrophages appeared to persist in the CTGF treated diabetic animals (*n* = 107 macrophages/20 fields) compared with the nontreated diabetic animals (*n* = 73 macrophages/20 fields) at day 14, although this did not reach statistical significance.

**Figure 2:**
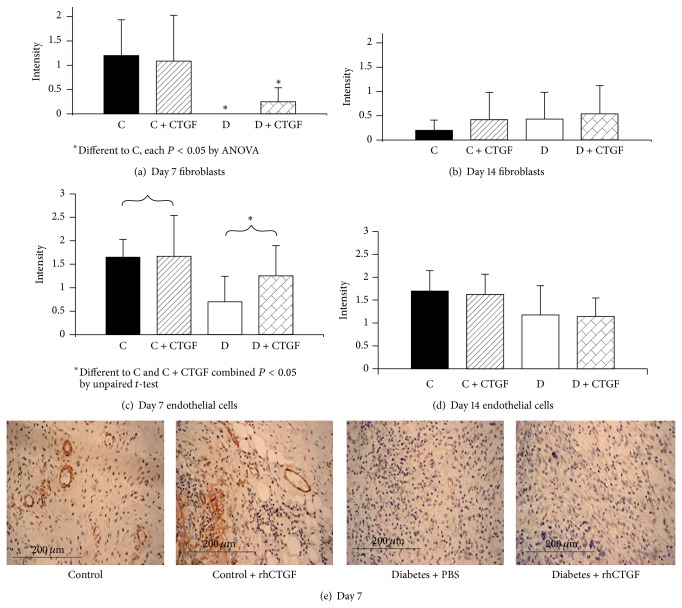
Wound *α*-smooth muscle actin staining in fibroblasts and endothelial cells. (a) shows *α*-SMA staining intensity scoring in fibroblasts, in treated control animals compared with the two diabetic animal groups, at day 7; ^*^ *P* < 0.05 by ANOVA for each of the groups marked versus Control + CTGF. No such differences were observed at day 14 in (b). In (c) increased *α*-SMA staining in endothelial cells was observed at day 7 in control animals (treated and untreated combined) compared with diabetic animals (treated and untreated combined); ^*^ *P* < 0.05 by unpaired *t*-test. No such differences were observed at day 14 (d). Results are expressed as mean ± SEM. Representative images at day 7 are shown in (e).

Intensity of staining by *α*-SMA, a marker of activated fibroblasts and mature vascular blood vessel cells, was measured at days 7 and 14. The fibroblast *α*-SMA in the nondiabetic rat wounds was higher (Figures 2(a) and 2(b)) than both the treated and untreated diabetic animals, indicating a relative lack of activated fibroblasts in the diabetic wounds by ANOVA (*P* < 0.05). Increased *α*-SMA staining in endothelial cells was observed at day 7 in control compared with diabetic animals, regardless of treatment (*P* < 0.05) (Figures 2(c) and 2(d)). The CTGF topical treatment of diabetic mice trended at day 7 to higher *α*-SMA staining score in fibroblasts (Figure 2(a)) and endothelial cells (Figure 2(c)) compared with scores in untreated diabetic mouse although on each occasion this did not reach statistical significance. Representative images of *α*-SMA staining are shown in Figure 2(e).

**Figure 3:**
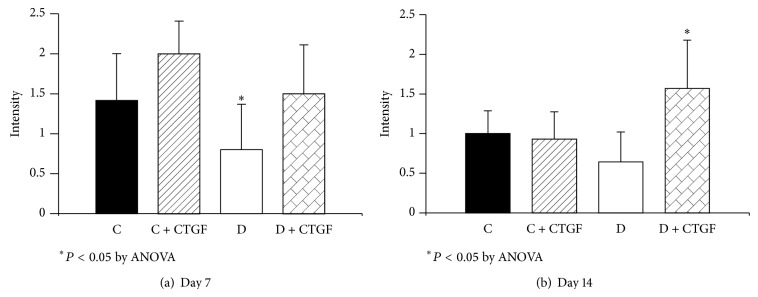
Wound collagen IV staining. (a) shows collagen IV staining was lagging in untreated diabetic wounds at day 7; ^*^ *P* < 0.05 compared with Control + CTGF treated animals, by ANOVA. (b) shows at day 14 the CTGF treated diabetic animals showed significantly increased collagen IV compared with the other groups; ^*^ *P* < 0.05 by ANOVA. Results are expressed as mean ± SEM.

**Figure 4:**
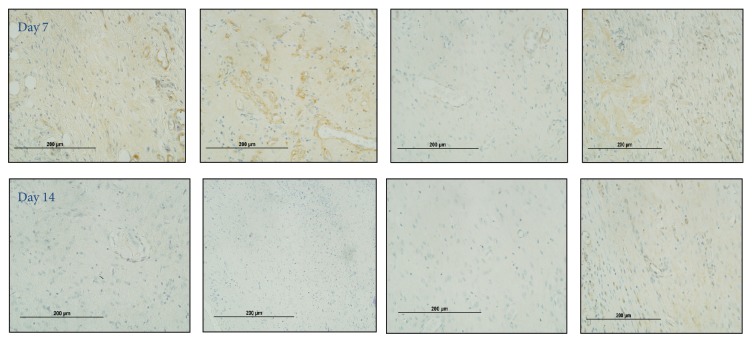
Wound collagen IV staining images. Representative images reflect that collagen IV is increased at day 14 in CTGF treated diabetic rodent wounds relative to the other groups and remained at a level similar to that observed at day 7.

Collagen IV staining was lower in untreated diabetic wounds at day 7 and was increased by rhCTGF (*P* < 0.05) (Figure 3(a)). At day 14 CTGF treated diabetic wounds showed significant increases in wound collagen IV compared with untreated diabetic wounds (*P* < 0.005) (Figure 3(b)). Representative collagen IV images for days 7 and 14 from rats within respective groups are shown in Figure 4.

### 3.4. Endogenous CTGF Changes in Human Diabetic Foot Ulcer Wound Fluid

**Figure 5:**
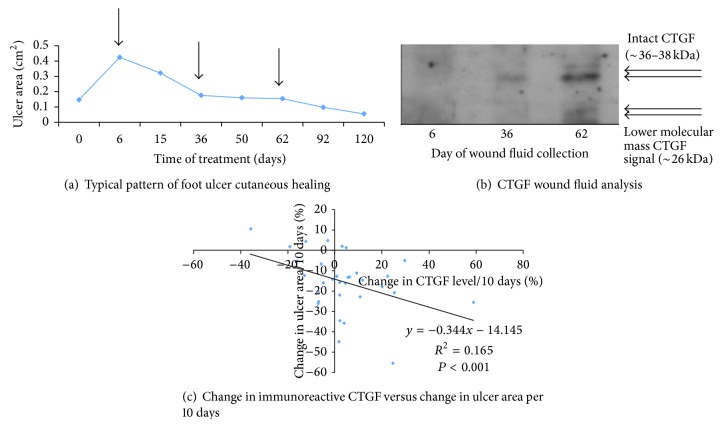
Endogenous ulcer fluid CTGF and diabetic foot ulcer healing. In (a) ulcer area versus time is shown for a typical small hallux plantar ulcer, with the arrows indicating sampling of postdebridement ulcer fluid. The ulcer fluid in (a) was analyzed by Western Immunoblot to detect CTGF immunoreactivity under nonreducing conditions, as shown in (b). Both intact monomeric CTGF and a lower molecular mass ~26 kDa CTGF are observed. Images such as those in Figure 5(b) were quantitated by densitometry, to compare change in ulcer fluid CTGF immunoreactivity versus change in ulcer area, each across 10 days. In (c), CTGF was found to increase as ulcer area reduced (*R* ^2^ = 0.165; *P* < 0.001).

Postdebridement wound fluid samples obtained from people with type 2 diabetes who had foot ulcers were analyzed for CTGF by Western immunoblot analysis, and wound area was determined at each visit based on acetate tracings.  Figure 5(a) shows a typical cutaneous wound area profile in a patient with a neuropathic hallux plantar ulcer, with times (arrows) when ulcer fluid was collected and then analyzed the following serial debridements. The respective Western immunoblot analysis for CTGF in the ulcer fluid is shown in Figure 5(b). CTGF was detected as a predominant intact ~36–38 kDa series of molecular mass bands, typical of known intact CTGF glycoforms [24]. In some immunoblots, there was also lower molecular mass signal at ~26 kDa (Figure 5(b)) as previously observed in biological fluids [18].

The group data (*n* = 32 study subjects) relating ulcer healing rate to rate of change in CTGF in ulcer fluid across an average time period of 10 days showed that an increase in CTGF levels correlates highly significantly with wound healing rate, indicated as percentage reduction in ulcer area per 10 days (Figure 5(c)); *r* = 0.406; *P* < 0.001. While there was quite marked interindividual variation in the data, on average a 10% increase in CTGF levels in wound fluid across 10 days correlated with ~17.6% improvement (i.e., a reduction) in ulcer area (Figure 5(c)).

## 4. Discussion

In diabetic wounds, healing is known to be impaired by several abnormalities including prolonged inflammation, impaired neovascularisation, decreased synthesis of collagen, and defective macrophage functions [6]. CTGF has been shown* in vitro* to be important in wound healing [25] and to accelerate healing rate of burns in rhesus monkeys [13].

The current study shows that topical application of CTGF improves wound healing in a diabetic rodent model of full thickness cutaneous wound healing. The main end point, rate of epithelial closure as a percentage of original wound size, was accelerated in the group of diabetic rats that were treated with CTGF. This was demonstrated by the serial measurement of wound area over time. Occlusion of the wounds using a semipermeable film dressing “Tegaderm” further validates this model as occlusion enabled more accurate visualisation of the wound as no scab or foreign matter was able to distort or contaminate the wound. Furthermore, it has been shown that, in diabetic rodent models, the film dressing exerts a “splinting” effect to the wound margins and contraction, thus promoting healing through reepithelialization [26] which better approximates human wound healing, rather than through contraction which predominates in nonsplinted rodent wounds.

A lack of detectable effect of CTGF on control wounds indicates that CTGF is able to normalise certain deficits found within diabetic wounds without affecting healing in normal wounds. It is possible that CTGF is able to improve wound healing through augmentation of cellular chemotaxis and mitosis and/or through upregulation of related mediators of CTGF such as TGF-*β*. These actions may attenuate the persistent inflammation which is detrimental within the diabetic wound. The monkey cutaneous burns model previously published that also responded to rhCTGF topically with ulcer closure [13] also has a proinflammatory environment, suggesting that rhCTGF may be working through such mechanism in cutaneous wounds.

The beneficial effect of the rhCTGF therapy in terms of epithelial closure appeared to occur quite early in wound healing. A CTGF effect was observed in diabetic wounds, which was statistically significant by day 7 after wounding. In this context it is notable that CTGF is known to induce macrophage chemotaxis [27]. In addition, application of macrophages or stem cells onto diabetic rodent wounds has been shown to accelerate wound healing and epithelial closure [28]. In the current work while it appears that macrophage number was increased in wounds treated with CTGF, this was not statistically significant. It may be that some of the CTGF effect was through induction of macrophage presence in diabetic wounds: increased macrophage infiltration observed within the CTGF treated diabetic wounds at day 7 cosegregated with accelerated healing of these wounds. In turn, macrophages upregulate healing through their inflammatory and reparative phenotypes and the balance between inflammatory and repair macrophages is crucial for successful healing [29]. Inflammatory macrophages synthesize a plethora of growth factors which in turn attract fibroblasts and endothelial cells and promote their proliferation [30]. Reparative macrophages also support ECM remodeling [29].

The finding that Young's modulus was lower in the diabetic animals than the controls is in keeping with recent literature [31]. Interestingly Young's modulus was increased by *t*-test (*P* < 0.05) in animals that were treated with CTGF compared to untreated animals, regardless of diabetes status. It is likely that the observed increase in collagen IV seen in earlier experiments after CTGF treatment is contributing to the greater stiffness of the wound sites. Collagen is known to contribute to wound strength and therefore increased stiffness and strain in CTGF treated wounds is probably attributable to increased collagen IV in these tissues. These observations are consistent with similar findings for other growth factors such as PDGF, where treatment induced collagen IV and increased strength in corneal tissue [32] and in preclinical models of diabetic ulceration [33].

Collagen IV was markedly increased in CTGF treated diabetic ulcers compared with both untreated diabetic ulcers and controls. This formation shows progression of the wound to remodeling and therefore end stage repair. Collagen IV peptides have been shown to promote cell adhesion and migration in corneal epithelial cells [34]. The overall collagen protein content of a wound is determined by the balance between collagen production and absorption [7], and MMP 9 is a key regulator of collagen IV [6]. The direct effects of CTGF on MMP activity have not been extensively studied. However recently we reported that CTGF upregulates the expression of TIMP-1, an MMP inhibitor, in mesangial cells [19], and Wang et al. reported earlier that rhCTGF induced TIMP-1 and TIMP-2 in porcine skin [35]. It is therefore possible that increased collagen content in the diabetic wound is attributable to CTGF mediated upregulation of TIMPS and corresponding inhibition of MMP 9.

CTGF treatment increased expression of smooth muscle actin in both fibroblast and endothelial cells. This finding is similar to the enhanced expression of *α*-SMA seen in subcutaneous rat fibroblasts following treatment with transforming growth factor-*β*1 [36, 37]. Both endothelial cell and fibroblast expression of *α*-SMA are robust predictors of healing [38]. Increased *α*-SMA in these cells is indicative that the wound is progressing from the inflammatory phase and that contraction: remodeling and closure are occurring at an accelerated rate. While some recent reports have implicated CTGF/CCN2 in postwound scar formation [39, 40], we did not observe macroscopic excessive scarring in the treated CTGF/CCN2 wounds, although the planned limited study duration may have prevented optimal detection of such a change. This lack of late time point assessment is a limitation of the current study. As the wound breaking strength data show no marked change in final strain after CTGF treatment of wounds it is likely that fibrosis is not induced significantly in this model by rhCTGF. However future studies in diabetic cutaneous wounding will be required to formally address that issue of any potential late histological fibrosis induction by rhCTGF, which was not the primary focus of the current research.

This is the first time that changes in endogenous CTGF/CCN2 have been reported in human diabetic foot ulcers or related ulcer fluid. Across a series of samples in 32 study subjects we have found that as immunoreactive CTGF increases in the postdebridement ulcer fluid, the ulcer demonstrates signs of healing. CTGF is known to be induced during wounding of human skin [41, 42]. This data supports the concept that CTGF may have a role in ulcer healing in diabetes, and combined with the rodent data described in this paper and our previous data in a primate non-human (baboon) model of diabetic wounding [18], it provides greater rationale for studying CTGF as therapy in diabetic foot ulcers. The correct dosing and timing schedule as well as whether CTGF should be used alone, in combination with other growth factors [43] such as in matrix [44] or protease inhibitors [45], or in expression vectors, remains to be defined.

## Figures and Tables

**Table 1:** Wound closure data in diabetic and nondiabetic (control) rats, each treated with topical rhCTGF or PBS alone.

Wound closure (% of initial wound size)					
Group	Day 1	Day 3	Day 7	Day 10	Day 14
C + PBS alone	85.7 ± 1.5	57.7 ± 2.1	25.2 ± 0.9^*^	18.0 ± 0.6	13.0 ± 0.4
DM + PBS alone	92.8 ± 1.6	61.7 ± 1.7	31.0 ± 1.1	20.7 ± 0.8	15.9 ± 0.5
C + CTGF	81.7 ± 1.4^*^	52.2 ± 1.8^*^	25.6 ± 0.9^*^	16.5 ± 0.4	13.2 ± 0.3^*^
DM + CTGF	85.4 ± 1.4	57.5 ± 2.2	28.4 ± 1.2^*^	16.1 ± 0.7^*^	13.6 ± 0.4^*^

Results are shown as mean ± SEM. ^*^ *P* < 0.05 versus the same day diabetic wounds + PBS (DM + PBS alone), by ANOVA. The main finding was that ulcer area was significantly reduced on days 7, 10, and 14 in the CTGF treated diabetic group (D + CTGF) compared with the non-rhCTGF treated diabetic group (D + PBS alone).

**Table 2:** Wound breaking strength data in all groups.

	Young's modulus	Tear strength	Tear strain	Ultimate strength	Final strain
C + PBS alone	0.93 ± 0.09	0.56 ± 0.06	1.17 ± 0.10	1.06 ± 0.01	3.45 ± 0.22^*^
DM + PBS alone	0.87 ± 0.13	0.46 ± 0.05	0.71 ± 0.06	0.81 ± 0.13	2.20 ± 0.16
C + CTGF	1.21 ± 0.11	0.53 ± 0.05	1.04 ± 0.10	0.89 ± 0.06	3.08 ± 0.27^*^
DM + CTGF	1.01 ± 0.17	0.53 ± 0.05	0.87 ± 0.18	0.86 ± 0.10	2.32 ± 0.22

Data are mean ± SD; each ^*^ *P* < 0.05 versus DM + PBS, by ANOVA.

**Table 3:** Demographics of patients with diabetes who had postdebridement foot ulcer fluid analyzed.

Patient demographics	
Study subject number (*n*)	32
Age (years)^*^	64.1 ± 1.8
Diabetes duration (years)	17.6 ± 1.9
Gender (% male)	75
Ulcer type	
Neuropathic only	29
Neuroischemic	3

^*^All data are mean ± SD.
